# Experimental evolution of *Staphylococcus aureus* in macrophages: dissection of a conditional adaptive trait promoting intracellular survival

**DOI:** 10.1128/mbio.00346-24

**Published:** 2024-04-29

**Authors:** Joana Alves, Manouk Vrieling, Natalie Ring, Gonzalo Yebra, Amy Pickering, Tomasz K. Prajsnar, Stephen A. Renshaw, J. Ross Fitzgerald

**Affiliations:** 1 The Roslin Institute, University of Edinburgh, Easter Bush, Midlothian, Edinburgh, United Kingdom; 2 Florey Institute, Bateson Centre and Division of Clinical Medicine, School of Medicine and Population Health, Sheffield, United Kingdom; MedImmune, Gaithersburg, Maryland, USA

**Keywords:** *Staphylococcus aureus*, antibiotic resistance, macrophages, evolution

## Abstract

*Staphylococcus aureus* is a major pathogen associated with important diseases in humans and animals. Macrophages are a key component of the innate immune response to *S. aureus* infection and play a major role in disease outcomes. To investigate the adaptive evolution of *S. aureus* in response to macrophages, we developed an experimental infection assay. *S. aureus* strains representing major human epidemic clones were passaged many times in a macrophage cell line, accumulating mutations in an array of genomic loci. Phenotypic analysis revealed the emergence of a lineage exhibiting increased survival in macrophages and human blood, and resistance to vancomycin. The evolved lineage exhibited a previously undescribed small colony variant (SCV) phenotype characterized by hyper-pigmentation, which resulted from a missense mutation in *rsbW*. Notably, the novel SCV was a conditional adaptive trait that was unstable in nutrient-replete conditions *in vitro*, rapidly converting from hyper-pigmented SCV to a non-pigmented large colony variant via spontaneous *sigB* deletion events. Importantly, we identified similar deletions in the genome sequences of a limited number of clinical *S. aureus* isolates from public databases, indicating that related events may occur during clinical infection. Experimental infection of zebrafish did not reveal a difference in virulence between parent and novel SCV but demonstrated an *in vivo* fitness cost for the compensatory *sigB* deletion events. Taken together, we report an experimental evolutionary approach for investigating bacterial innate immune cell interactions, revealing a conditional adaptation that promotes *S. aureus* survival in macrophages and resistance to vancomycin.

**IMPORTANCE:**

*Staphylococcus aureus* is an important human bacterial pathogen. The host response to *S. aureus* involves the production of innate immune cells such as macrophages which are important for fighting infection. Here we report a new model of experimental evolution for studying how *S. aureus* can evade killing by macrophages. We identified a novel adaptive phenotype that promotes survival in macrophages and blood and resistance to antibiotics. The phenotype is lost rapidly upon growth in nutrient-rich conditions via disruption of the alternative sigma factor sigB, revealing a conditional niche-specific fitness advantage. Genomic analysis of clinical isolates suggests similar adaptations may occur during human infections. Our model may be used broadly to identify adaptations of *S. aureus* to the innate immune response.

## INTRODUCTION

*Staphylococcus aureus* is an important global pathogen, responsible for life-threatening infections in humans and major economic losses in livestock industries (1–3). *S. aureus* is a constituent of the normal microbiota but can cause an array of infections, ranging from superficial skin and other soft tissue infections to necrotizing pneumonia, infective endocarditis, and septicemia (4). This range of clinical manifestations is enabled by the production of an extensive repertoire of virulence factors (5) allied to a complex regulatory system by which *S. aureus* adapts to different challenges within the host (6). *S. aureus* readily undergoes adaptive evolution in the face of new challenges, facilitating survival and clonal expansion (7, 8). Importantly, *S. aureus* readily develops resistance to the majority of available antimicrobial agents, including the last-resort antibiotic, vancomycin (9). The spread of multidrug-resistant clones and the high mortality associated with invasive methicillin-resistant *S. aureus* infections (10) means that the development of new therapies is urgent. An understanding of the key host-pathogen interactions and bacterial adaptations in response to the host immune system could reveal new targets for infection control and aid the design of new therapeutics.

Macrophages play a key role in controlling bacterial burden and limiting *S. aureus*-associated mortality in different *in vivo* models (11, 12). However, *S. aureus* has developed strategies to evade or manipulate macrophages to its advantage (13). Subpopulations of infecting bacteria can survive and multiply inside mature phagosomes for extended periods (12, 14), by-passing killing mechanisms (15) before migrating to the cytoplasm, inducing host cell death and escaping to the extracellular milieu (16–18). In addition, the macrophage intracellular niche provides an environment that selects for the evolution of *S. aureus* small colony variants (SCVs) and cells with a persister phenotype, highly associated with antibiotic tolerance (19, 20). SCV forms characteristic small colonies on nutrient-rich agar due to their reduced metabolic rate, have lower levels of toxin expression, increased expression of adhesins, and are often auxotrophic for menadione, hemin, or thymidine (21). Several different mechanisms of SCV formation have been described involving global regulators (like *sigB*, *sarA,* and *agr*) (22, 23), ATPases, and non-coding RNAs (24, 25). In addition, large-scale reversible intra-chromosomal rearrangements have been linked to SCV formation (26, 27). Importantly, SCV phenotypes are often dynamic and many can revert to fully virulent wild-type forms in the extracellular milieu (28).

Intra-host and adaptive laboratory evolution studies have been employed to identify *S. aureus* mechanisms of tolerance and resistance to antibiotics (29–31), mechanisms of host-adaptation (32), and adaptation to different human tissues (33, 34). Here we designed an *in vitro* adaptive laboratory evolution model to study evolutionary pathways for adaptation of *S. aureus* to the macrophage intracellular environment. We identified an evolved variant of *S. aureus* that had enhanced macrophage intracellular survival, increased survival in blood, and vancomycin-intermediate resistance phenotype. It had a novel SCV phenotype associated with hyper-pigmentation, which readily transitioned to a large unpigmented colony type after culture in nutrient-replete conditions via large deletions in *sigB*. Similar deletions were identified in clinical isolates in *S. aureus* genome databases, indicating that this might be a conserved mechanism for the reversion of SCV phenotype by *S. aureus*. The identification of a conditional adaptive phenotypic trait that is lost during *in vitro* culture highlights the power of models of experimental evolution for understanding key host-pathogen interactions relevant to infection.

## MATERIALS AND METHODS

### Bacterial strains and growth conditions

N315 is a methicillin-resistant *S. aureus* strain isolated in 1982 from the pharyngeal smear of a Japanese patient (35) and SH1000 is a laboratory *rsbU*-repaired strain from the phage-cured 8325–4 (36). All strains utilized in the study were kept frozen in tryptic soy broth (TSB, Oxoid) with 25% (vol/vol) glycerol and were routinely cultured in TSB at 37°C with shaking. *Escherichia coli* strains were cultured in Luria-Bertani broth (LB, Sigma) at 37°C with shaking.

### Macrophage culture and differentiation

The THP-1 human monocytic cell line (kindly gifted by Prof. David Hume) was maintained in RPMI-1640 medium (Sigma) supplemented with 10% (vol/vol) heat-inactivated fetal bovine serum (FBS, Life Technologies) and GlutaMax (Gibco) at 37°C, 5% CO_2_. For each infection experiment, THP-1 monocyte cells were differentiated into macrophages by treatment with 200 nM phorbol 12-myristate 13-acetate (PMA, VWR) for 48 h or 72 h at the concentration of 1 × 10^5^ cells per well in 96-well plates (Nunc). Media was replaced with media without PMA for 24 h prior to infection. Cell differentiation following PMA stimulation was monitored over time by microscopic observation of cell morphology and plastic adherence properties.

### Bacterial adaptive laboratory evolution of *S. aureus* in THP-1 macrophages

**Fig 1:**
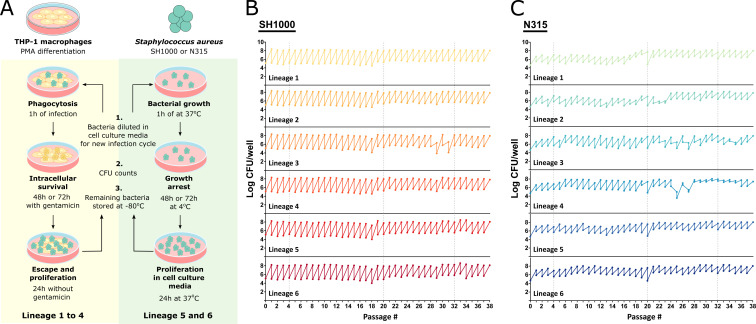
An experimental model of *S. aureus* evolution in human macrophages. (**A**) Schematic representation of the long-term *S. aureus* THP-1 macrophage infection protocol. For each infection cycle, THP-1 macrophages were differentiated by PMA stimulation (as described in Materials and Methods) and infected with Lineages 1–4 of N315 or SH1000 for 1 h. Bacteria were kept under intracellular selective pressure by incubating the culture with a low dose of gentamicin for 48 h or 72 h. Antibiotics were then removed and the bacteria were allowed to escape and multiply in the extracellular space for 24 h. As a control, N315 and SH1000 Lineages 5 and 6 were grown in macrophage culture media alone. The bacteria were incubated at 37°C for 1 h and then incubated at 4°C for 48 h to 72h to mimic the growth restriction of the antibiotic step of Lineages 1–4. The culture was then moved to the 37°C incubator for 24 h. At the end of the cycle, the cell culture media was collected and used for a new infection cycle, bacterial quantification, and colony analysis in TSA and blood agar plates, and the remainder was stored at −80°C in glycerol. Figure made using Servier Medical Images. (**B and C**) Amount of bacteria used for infection at the beginning of each infection cycle and recovered at the end of each cycle, after 24 h at 37°C without antibiotics. Lineages 1–4 evolved in the presence of THP-1 macrophages and Lineages 5–6 in macrophage culture media alone. Total of 38 cycles.

For the first cycle of infection, one colony of *S. aureus* strains SH1000 and N315 was cultured overnight in TSB at 37°C with shaking. The bacteria cells were sub-cultured to exponential phase (OD_600nm_ ≈ 0.6), washed and suspended in macrophage cell culture media, and resuspended to 1 × 10^6^ bacteria/mL. Throughout the serial passaging assay, THP-1 macrophages were infected with 100 µL of bacterial culture, and the plates were centrifuged for 5 min at 300 × *g* to promote contact with the macrophages. After 1 h of incubation at 37°C in a 5% CO_2_ atmosphere, the media was replaced with media with 100 µg/mL of gentamicin (Sigma) for 30 min to kill all extracellular bacteria. Media was then removed, cells were washed with macrophage media and maintained at 37°C in a 5% CO_2_ atmosphere for 48 h or 72 h in media with 10 µg/mL of gentamicin. This dose was used to kill extracellular *S. aureus*, maintain the intracellular selective pressure, and minimize the amount of antibiotics in the macrophage intracellular environment (37). At this point of the protocol, the number of bacteria that could be recovered from inside of the macrophages was too low to allow a new re-infection cycle (Fig. S1). For that reason, the antibiotics were removed from the culture for 24 h to allow bacterial replication in the cell culture media. At the end of this timepoint, sufficient bacterial numbers were reached in the media (Fig. S1; Fig. 1), and macrophages were killed in the co-culture. Next, the media was collected from the cultures, and 20 µL volumes were used for serial dilution in macrophage media for CFU quantification on TSA plates and a new re-infection cycle. The remaining volume was stored in 25% glycerol in TSB at −80°C. As a control, *S. aureus* was cultured in the macrophage media on the same plates and at the same concentration used for THP-1 infection. The plates were kept at 37°C in 5% CO_2_ for 1 h, in parallel to the internalization stage of the macrophage infection, and kept at 4°C when the THP-1 culture was maintained in the presence of antibiotics (for 48 h or 78 h). The bacteria were then transferred to the 37°C incubator for 24 h and subjected to the same dilutions and storage treatment as described above for the macrophage cultures (Fig. 1A). All the re-infections were performed from bacteria recovered from cultures maintained at 37°C, 5% CO_2_ for 24 h without antibiotics, except passages 5 and 21, which were infected by bacteria grown in TSB from frozen stocks of passage 4 and 20, respectively. In total, six lineages were started from the initial parental sample for strains SH1000 and N315, Lineages 1–4 were evolved in the macrophage culture, while Lineages 5 and 6 were grown in macrophage culture media alone.

### Whole-genome sequencing and comparative analysis

DNA was sequenced from the two parental strains, SH1000 and N315, and from six colonies from the passaged samples from each of the six lineages at passages 4, 20, and 32. An extra set of six samples was also sequenced from N315 lineage 3 at passage 36. Thus, 30 samples were sequenced per strain (five per lineage) at each passage point, 90 in total for SH1000 and 95 in total for N315. The isolates were grown on TSA plates and sent to MicrobesNG (Birmingham, UK) for Illumina MiSeq sequencing.

### Identification of genomic variants: SNPs, deletions, and insertions

Raw fastq reads were trimmed using Trimmomatic (v0.39, 38). Variant calling was conducted with Snippy (v4.6.0, 39), using the parental sequences of SH1000 and N315 as the reference genomes for each set of samples, respectively.

### Growth curves

*S. aureus* isolates were grown overnight in TSB at 37°C with shaking. A subculture of the overnight growth was made in macrophage media (RPMI supplemented with 10% FBS and GlutaMax) in a volume of 200 µL in 96 flat bottom well-plate (Nunc) and kept at 37°C with shaking (200 rpm) in a CLARIOstar plate reader (BMG labteck). Optical density at 600 nm was recorded every 10 min for 10 h.

### THP-1 macrophage infections

*S. aureus* isolates were grown overnight and then sub-cultured to exponential phase (OD_600 nm_ ≈ 0.6) in TSB at 37°C with shaking. The bacteria were washed and suspended in macrophage cell culture media 1 × 10^6^ bacteria/mL. THP-1 macrophages were infected with 100 µL of bacterial culture and the plates were centrifuged for 5 min at 300 × g to promote contact with the macrophages. After 1 h of incubation at 37°C in a 5% CO_2_ atmosphere, the media was replaced with media with 100 µg/mL of gentamicin (Sigma) for 30 min to kill all extracellular bacteria. The media was then removed, cells were washed with macrophage media and maintained at 37°C in a 5% CO_2_ atmosphere for 72 h in media with 10 µg/mL of gentamicin. Bacterial escape from the macrophage intracellular environment and growth in the media was evaluated 6 and 24 h after the removal of the antibiotics. To evaluate the bacterial levels at the different time points, macrophages were lysed with 0.1% Triton X-100.

To evaluate intracellular survival after a macrophage infection cycle, bacteria were recovered from a THP-1 culture 24 h after antibiotics removal and used to infect a new THP-1 macrophage culture. After 1 h of infection, the media was replaced with media with gentamicin 100 µg/mL (Sigma) for 30 min to kill all extracellular bacteria. Antibiotics were removed by washing the culture three times with phosphate-buffered saline (PBS) and macrophages were lysed with 0.1% Triton X-100. Bacterial survival was evaluated by comparing bacterial levels after 72 h inside the macrophages to bacterial levels after 1 h of phagocytosis.

### Whole blood infection

*S. aureus* isolates were grown overnight in TSB at 37°C with shaking. Fresh human whole blood (150 µL) was infected with 50 µL of bacteria at 2 × 10^7^ CFU/mL in PBS. Infection was maintained at 37°C with orbital shaking at 200 rpm for 60 min. Blood was lysed in 0.1% (vol/vol) TritonX-100 (Sigma) in PBS and viable bacteria counts were determined.

### Vancomycin resistance

*S. aureus* isolates were grown overnight in TSB at 37°C with shaking. A subculture of the overnight was made in TSB in the presence of different concentrations of vancomycin (Sigma) in 96 flat bottom well-plate (Nunc) and kept at 37°C with shaking (200 rpm) in a CLARIOstar plate reader (BMG labteck). Optical density at 600 nm was recorded every 10 min for 24 h. Growth curves were plotted using GraphPad Prism v7 and the area under the curve (AUC) was calculated using the same software. The percentage of AUC was calculated by comparing the AUC of the grown in the presence of each vancomycin concentration with AUC in the absence of antibiotics (control).

### Long-read sequencing genome assembly, genome arrangement comparison, and base modification and motif detection

High molecular weight gDNA was extracted from N315 parental, N315 L3 passage 32 orange, and N315 L3 passage 32 white colonies. Bacteria were grown overnight on TSA plates, and then gDNA was extracted using Qiagen’s MagAttract HMW DNA Kit (Qiagen, Manchester, UK) according to the manufacturer’s instructions for Gram-positive species, using 20 µL of lysostaphin (10 mg/mL) instead of lysozyme. The extracted HMW gDNA was sent to the Centre for Genomic Research at the University of Liverpool, UK, where long-read sequencing, base modification analysis, and motif detection were conducted on the Pacific Biosciences (Pacific Biosciences, Menlo Park, CA, USA) Sequel system.

Over 1,000 × coverage was produced for each sample sequenced; Filtlong (v0.2.0, 40) was used to filter each read set to 560 Mb (around 200 × coverage) using the longest and highest quality reads. The filtered reads were then assembled with Flye (v2.7b-b1526, 41), using the options --pacbio-raw and --genome-size 2.8 m. The arrangement of the assembled genomes and plasmids was then compared using progressiveMauve (v16Sep2015 [42])

The motif sites detected during sequencing were compared sample-to-sample, using a score cut-off of 200, to identify any differences.

### Construction of *corA* and *rsbW* mutants

Allele replacement mutagenesis of *corA* and *rsbW* was performed using the thermosensitive plasmids pIMAY (RRID:Addgene_68939) (43). For generation of the N315::*rsbW^L3^,* N315::*corA^L3^,* N315::*corA^L3^rsbW^L3^*, and N315 L3::*rsbW^WT^* mutants, *corA* and *rsbW* genes from N315 L3 or N315 WT isolates were amplified with the primers in Table S1. These primers amplified the gene of interest and the approximately 500 bp flanking region and have a complementary sequence to the multiple cloning site (MCS) of pIMAY. The genes were amplified using Q5 High-Fidelity DNA Polymerase (NEB), and the PCR product was purified using the Monarch PCR and DNA cleanup kit (NEB). Restriction digestion of the PCR products and plasmid was performed at 37°C for at least 2 h using the *Sac*I and *EcoR*I restriction enzymes (NEB). The digested plasmid was treated with Antarctic Phosphatase (NEB) and cleaned using the Monarch PCR and DNA cleanup kit before overnight ligation with T4 DNA Ligase (NEB) at a 3:1 molar ratio of insert:plasmid. Dialysis of the ligation reactions was performed using 0.025 µm filter circular discs (Millipore) before transformation into chemical competent*-E. coli* DC10B. All plasmid constructs were verified by Sanger sequencing (Eurofins) before purification using the Monarch plasmid purification kit (NEB). *S. aureus* competent cells were produced using a method outlined previously (43). The plasmids were concentrated using Pellet Paint co-precipitant (Novagen) and electrotransformed into electrocompetent *S. aureus* N315 WT or L3 orange from passage cycle 32, depending on the construct. The transformants were selected by incubation at 30°C in TSA with 10 µg/mL chloramphenicol. Once the plasmids were transformed into *S. aureus* at 30°C, growth at the restrictive temperature of 37°C was selected for integrants. OUT primers, located outside the flanking regions and genes of interest, were used to determine the success of the integration. For plasmid excision, colonies with positive integration were grown in TSB overnight at 28°C and plated into TSA with 1 µg/mL anhydrotetracycline. Allele exchange was confirmed in colonies that showed resistance to anhydrotetracycline and susceptibility to chloramphenicol using OUT primer PCR and sequencing the resultant fragment. In the case of the double mutant N315::*corA^L3^rsbW^L3^*, allelic exchange of the *rsbW* gene was performed as previously described in the single mutant N315::*corA^L3^*.

### Staphyloxanthin extraction

Bacterial strains were grown overnight with shaking at 37°C in TSB. Methanol extraction of carotenoid was performed as previously described (44) with the modification that 5 mL of the culture was harvested by centrifugation and cells were suspended in 1 mL of methanol. OD_465_ was measured in a spectrophotometer (SpectraMax ABS Plus).

### Zebrafish infection model

Infections were carried out as described previously (16). Briefly, inbred zebrafish embryos were provided from the UK Home Office-approved aquaria at the Bateson Centre, University of Sheffield. Embryos were incubated in E3 medium at 28.5°C and at 30 h post-fertilization were mechanically dechorionated and anesthetized by immersion in 0.02%(wt/vol) buffered tricaine (Sigma). Embryos were embedded in 3%(wt/vol) methylcellulose and injected individually with bacterial suspension of known concentration using pulled glass microcapillary pipettes into the yolk or the bloodstream. To recover bacteria, infected embryos were mechanically homogenized using a micropestle (Eppendorf) followed by serial dilution and plating out on solid BHI media followed by colony counting.

### Genomic analysis of clinical isolates from public databases

The Staphopia database (45) was used to investigate the presence of common mutations in clinical isolates. Annotated genomes, and their associated metadata, were downloaded for 45,885 *S*. *aureus* isolates (Table S2). MLST (v2.19.0 [46]) was used to assign sequence types (STs) to the annotated genomes, according to the *S. aureus* MLST scheme defined by PubMLST (47).

The lengths of the annotated *rsbW* and *sigB* genes were used as an initial proxy to identify potential non-synonymous mutations. Putative deletion-causing mutations were then manually confirmed using blastn to search for the *rsbW* and/or *sigB* (48). Some isolates possessed large predicted deletions, while in others *sigB* appeared to be located on a contig boundary, thus potentially giving the false appearance of a truncated gene. The presence or absence of deletions in these isolates was determined by downloading their raw reads using fasterq-dump (v.2.10.5) from the SRA toolkit and mapping the reads to the wild type *sigB* gene using bwa mem (v0.7.17 [49]) then manually inspecting the raw read support for the suspected deletion using Tablet (50).

For 132 isolates with confirmed *sigB* deletions, insertions, or early stop codons, and 118 randomly chosen, ST matched when possible, non-sigB-deficient isolates (Table S4), raw reads were downloaded using fasterq-dump, if not already downloaded for deletion verification. Snippy was then used to call variants in the entire *sigB* operon (*rsbU* to *sigB*), to compare to the SNPs identified in the passaged strains.

## RESULTS AND DISCUSSION

### Design of an experimental evolution model of *S. aureus* infection of macrophages

To examine the adaptive evolution of *S. aureus* during growth in human macrophages, we developed a model of successive passaging of *S. aureus* in THP-1 macrophages (Fig. 1 and Materials and Methods). In total, four lineages derived from a single colony of strains N315 and SH1000 were passaged in macrophage cultures (Lineages 1–4) and two lineages were passaged in cell culture media alone (Lineages 5 and 6). Considering *S. aureus* replication from 10^3^ bacteria approximately inside macrophages (Fig. S1) to 10^8^ at the end of the infection cycle (Fig. S1; Fig. 1), we estimate that after 38 infection cycles, each lineage had undergone the equivalent of a minimum of 630 generations. In the case of SH1000, the growth rate did not change significantly across cycles (Fig. 1B). However, we observed considerable variation in the number of recovered N315 during the infection protocol, and inoculums for reinfection were adjusted accordingly (Fig. 1C).

### Genomic diversification of *S. aureus* during sequential infections of human macrophages

**Fig 2:**
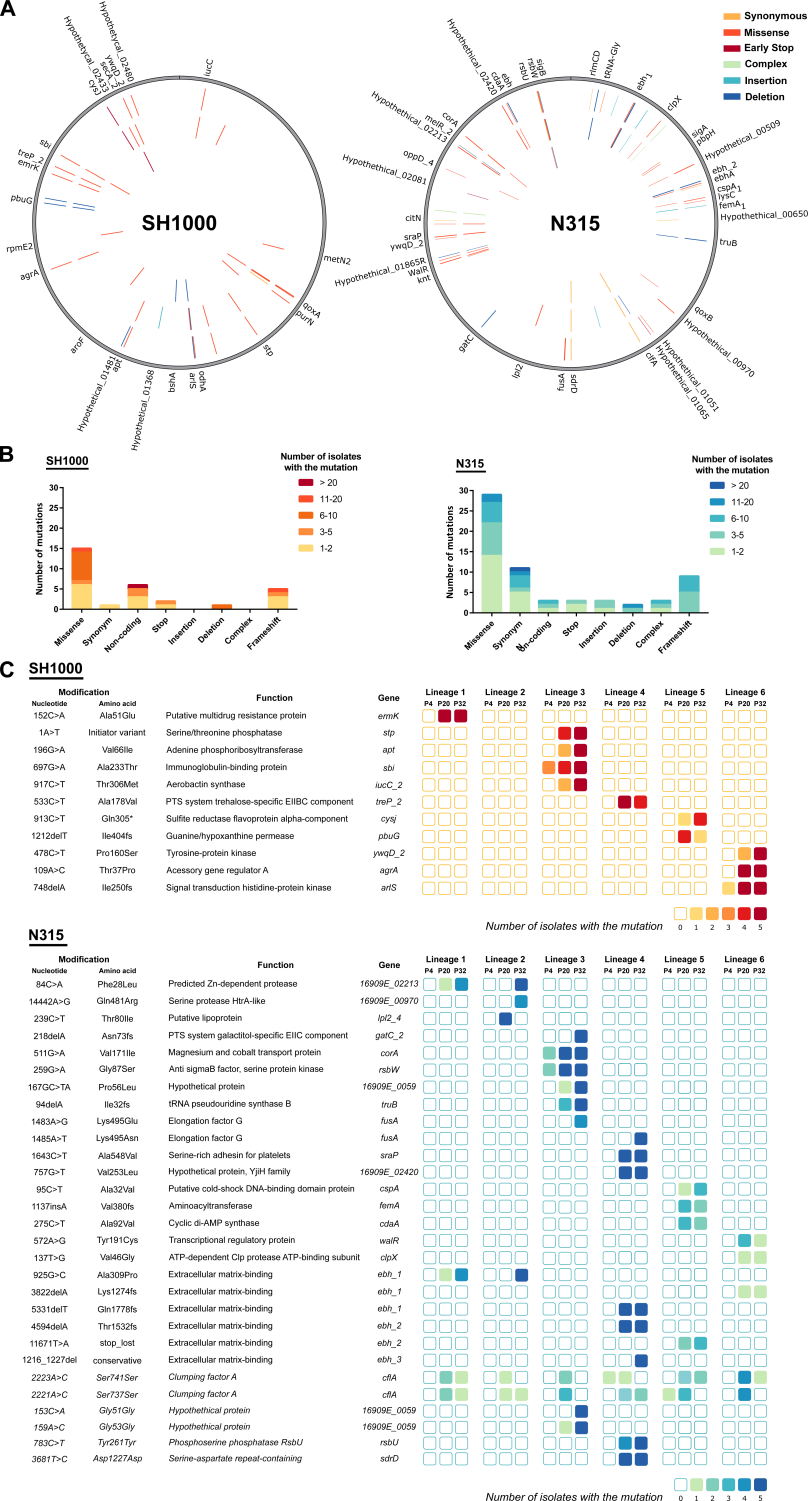
Mutations acquired during *S. aureus* sequential infections of macrophages. (**A**) Distribution of different types of mutations across the genomes and genes affected, colored by type of mutation. Mutations in isolates from passage cycles 4, 20, and 32 are plotted in the inner, middle, and outer circles, respectively. (**B**) Frequency of the types of mutations (from the 90 Illumina sequenced isolates per strain). (**C**) List of mutations found in more than three isolates in the passage cycle 4 (P4), 20 (P20), and 32 (P32), colored by the number of isolates with the mutation (from the five sequences per lineage, passage cycle, and strain). Synonymous mutations in italic.

To examine the diversification of *S. aureus* strains during the macrophage passaging experiment, we performed whole-genome sequencing (WGS) of five isolates from each of the lineages (Lineages 1–6) at infection cycle numbers 4, 20, and 32, representing a total of 180 isolates sequenced (90 from each strain). The lineages acquired single-nucleotide polymorphisms (SNPs), short indels, and deletions with a genome-wide distribution (Fig. 2A). In total, 70 single-nucleotide polymorphisms (SNPs) were identified among the 180 isolates including 8 in intergenic regions, 44 nonsynonymous, 12 synonymous, and 5 non-sense mutations, in addition to 14 indels leading to frameshifts (Fig. 2B). Many of the mutations are associated with determinants of host-pathogen interactions including cell envelope-associated proteins, proteases, and transporters (Fig. 2). A greater number of mutations were detected in isolates from the N315 lineage (*n* = 63) compared to the SH1000 lineage (*n* = 30) (Fig. 2), suggesting strain-dependent differences in the propensity for adaptive evolution. No mutations or affected genes were found to be shared between isolates from these two lineages. A limited number of non-synonymous mutations occurred in multiple lineages in parallel including the substitution 968801A > G in an intergenic region between *sspA* and *patA* in strain SH1000, while in N315 missense mutations in *16909E_02213* (predicted ZN-dependent protease) and in *ebh* occurred in isolates from passages 20 and 32 of Lineage 1 and isolates from passage 32 of Lineage 2 (Fig. 2D). Additional mutations in *ebh* occurred in Lineages 4, 5, and 6 of N315 (Fig. 2D). Ebh encodes an extracellular matrix (ECM)-binding protein involved in tolerance to transient hyperosmotic pressure and complement resistance (51, 52). Deletion or truncation of the *ebh* gene is associated with increased oxacillin and teicoplanin susceptibility (51, 52) but missense mutations in *ebh* genes have been widely reported previously, without a clear influence on bacterial phenotype (31, 53, 54).

Mutations in *fusA* encoding Elongation factor G, the target of the antibiotic fusidic acid, were identified in both Lineage 3 and Lineage 4 of N315 (Fig. 2D). Mutations in *fusA* frequently occur in *S. aureus* clinical isolates, causing a reduction in the affinity of fusidic acid for the Elongation Factor G-ribosome complex and consequent fusidic acid resistance (55, 56). A subset of these mutations has been linked to the development of a SCV phenotype (57). Of note, in a macrophage *E. coli* evolutionary study with a gentamicin incubation step, two lineages acquired the same *fusA* mutation, which was attributed to a possible growth compensatory mutation (58).

Synonymous mutations of *adrD*, *rsbU,* and in the hypothetical protein *16909E_0059* were maintained in the lineages once acquired (Fig. 2A and D). The maintenance of synonymous mutations could be driven by genetic drift or via selection acting on the stability of mRNA molecules affecting translation levels or influencing gene regulation by small RNAs (59, 60). Overall, a wide array of mutations occurred in different lineages that are predicted to impact bacterial fitness during co-infection with macrophages.

### Passaging of *S. aureus* in THP-1 macrophages selects for pathogenic variants

We asked whether the mutations identified were associated with a fitness advantage and compared the phagocytosis, macrophage intracellular survival, and escape of isolates from passages 4, 20, and 32 compared to parental strains. When the passaged isolates were grown in TSB prior to macrophage infection, no differences were detected in phagocytosis or intracellular survival compared to the parental isolates, for either SH1000 or N315 (Fig. S2A through F). The increase in the number of bacteria recovered for N315 lineages at passage 32 when antibiotics were removed from the media (Fig. S2F), is likely due to an increased growth rate in cell culture media alone (Fig. S3F) since the same growth pattern can be observed in lineages which evolved in the absence of THP-1 macrophages (Fig. S3D through F). Previously, it was reported that *S. aureus* sequentially grown in RPMI supplemented with LB Broth, evolved an increased growth phenotype (61).

**Fig 3:**
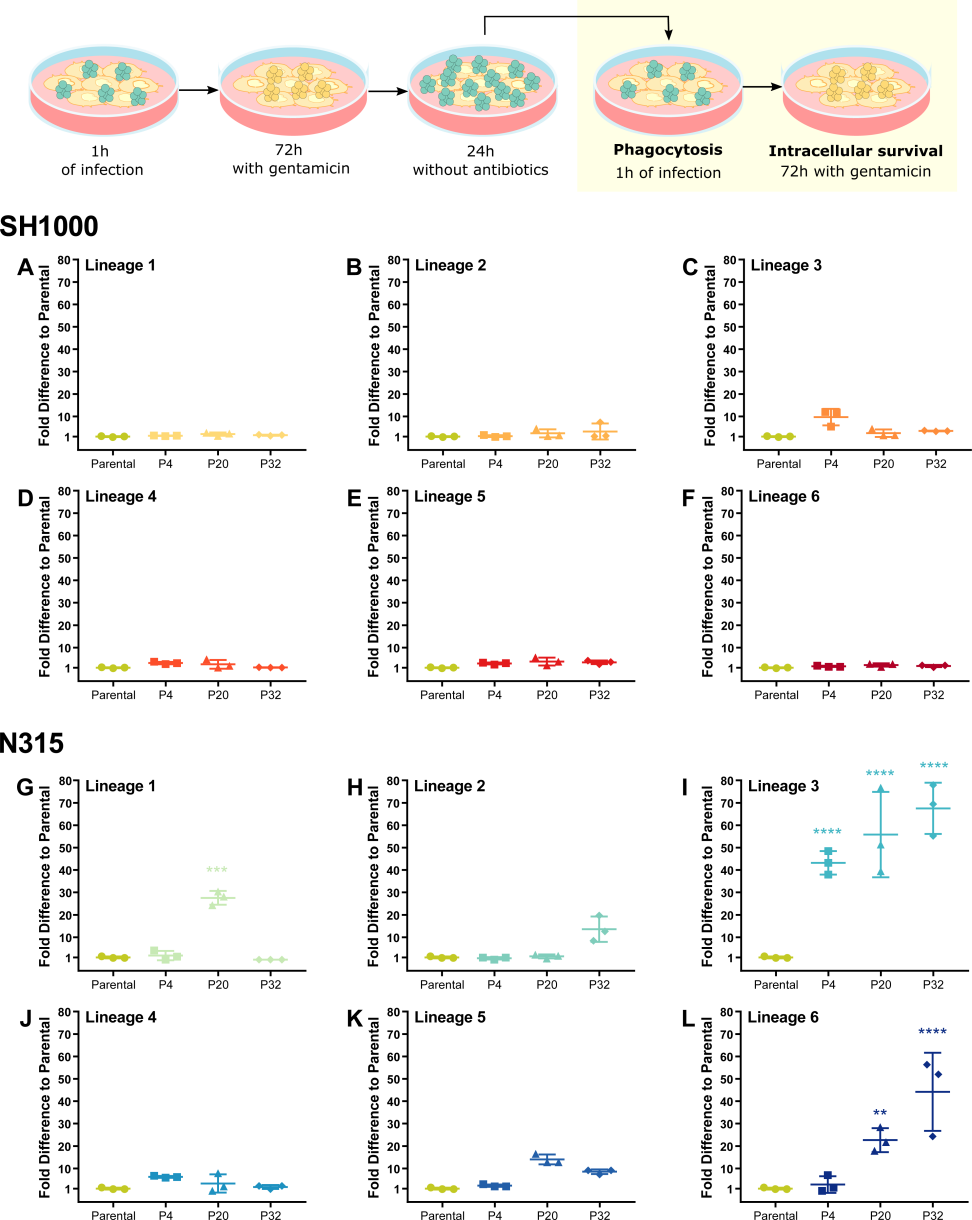
N315 Lineage 3 isolates exhibit increased fitness to survive THP-1 macrophage killing. THP-1 macrophages were infected with SH1000 (**A–F**) and N315 (**G–L**) parental strains and isolates from passage cycles 4 (P4), 20 (P20), and 32 (P20) from Lineages 1 to 6 after one cycle of THP-1 infection. THP-1 macrophages were infected with *S. aureus* at MOI 1 for 1 h, gentamicin was added for 72 h, and bacteria were allowed to escape and divide in the macrophage cell culture media prior to a new cycle of infection. New cycle: after 1 h of infection, the media was replaced with media with gentamicin. Bacterial survival was evaluated by comparing bacterial levels after 72 h inside the macrophages to bacterial levels after 1 h of phagocytosis. One-way ANOVA with Holm-Sidak’s multiple comparisons test to Parental. **P < 0.01, ***P < 0.001, ****P < 0.0001.

However, we did observe fitness differences between some N315 passaging isolates compared to the parental strain when the inoculum was prepared by passaging through the macrophage intracellularly followed by escape and replication in cell culture media prior to a new macrophage infection (Fig. 3). In particular, isolates from passages 4, 20, and 32 of the N315 Lineage 3 (N315 L3, Fig. 3I) and isolates passages 20 and 32 of the N315 Lineage 6 (N315 L6, Fig. 3L) exhibited an increased ability to survive in the macrophage intracellular environment when compared with the parental strain. No mutations or affected genes were shared between isolates of N315 L6 and N315 L3. One of the few mutations identified in isolates of N315 L6 was in *walR*, a gene of an essential two-component system involved in *S. aureus* cell division, metabolism, and virulence (62). However, we focused our attention on N315 L3 since it evolved during the THP-1 macrophage culture.

### *S. aureus* N315 Lineage 3 is a novel hyper-pigmented SCV with an unstable colony phenotype

**Fig 4:**
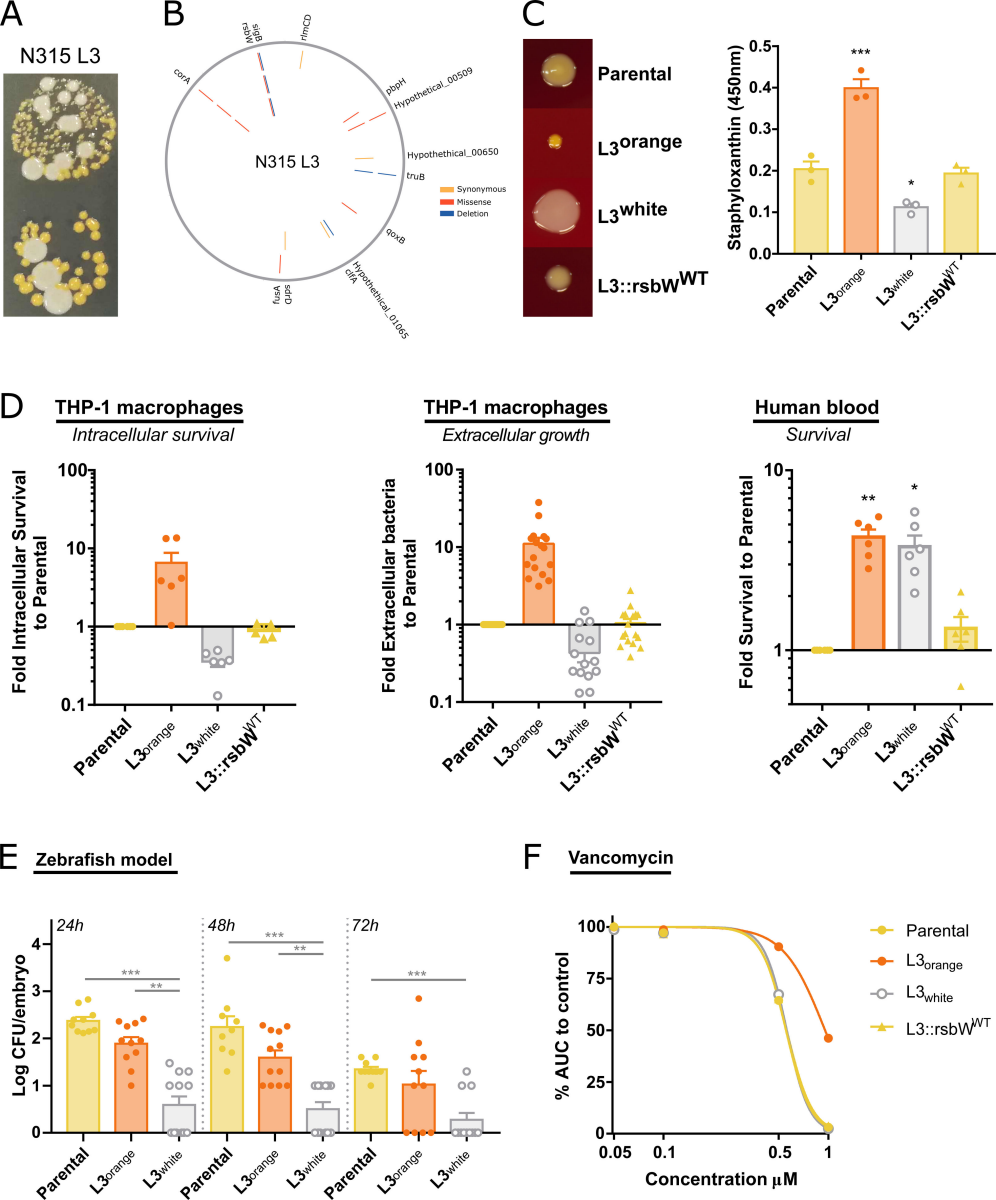
S. *aureus* N315 Lineage 3 is a hyper-pigmented SCV variant with an unstable colony phenotype. (**A**) Images of the unstable colony phenotype of N315 L3 passage cycle 32, with hyper-pigmented SCV and large white colonies. (**B**) Mutations identified in N315 L3 isolates from passage cycle 4 (inner circle), 20 (middle circle), and 32 (outer circle). Mutations in *corA* and *rsbW* were identified and maintained since passage cycle 4. (**C**) Reversion of *rsbW* mutation to WT in N315 L3 isolate reverts the colony phenotype. Photos of colony morphology from N315 parental, L3 orange, L3 white, and L3 with the *rsbW* mutation reverted to WT in THA incubated for 48 h at 37°C. Staphyloxanthin quantification after methanol extraction. (**D**) Bacteria grown overnight at 37°C in nutrient-rich media prior to infection. L3 orange and L3 white isolates showed different survival in human blood, intracellular survival (after 72 h with gentamicin), and extracellular growth in THP-1 cultures (after 24 h without antibiotics) to parental strain and L3::*rsbW*^WT^. (**E**) L3 white achieves lower bacterial levels than parental or L3 orange in a zebrafish embryo infection model. (**F**) L3 orange isolate has an intermediate vancomycin resistance phenotype. Bacteria were grown in TSB in the presence of increasing concentrations of vancomycin and the AUC of growth curves calculated. The AUC of each bacterial strain at the different vancomycin concentrations was normalized to growth curves in the absence of the antibiotic. Each point represents a biological replication. One-way ANOVA with Tukey’s multiple comparisons. *P < 0.05, **P < 0.01, ***P < 0.001.

Further characterization of N315 L3 revealed that isolates from as early as passage cycle 4 exhibited a colony phenotype that was distinguishable from the parental strain (pale golden colonies) and the other N315 lineages, presenting a highly pigmented (orange) SCV with delayed growth (24 h) on agar media (Fig. 4A and C). However, when Lineage 3 orange SCV is grown in solid nutrient-rich media, it converts to a colony phenotype that lacks hyper-pigmentation and has a restored growth rate. When the orange SCV was isolated and re-inoculated into nutrient-rich media and plated out, both orange and non-pigmented (white) colonies arose and subsequent subcultures of the white colonies indicated a stable phenotype (Fig. 4A and C). The unstable phenotype of the orange SCV is a classical characteristic of SCV, although it is typically described as a reversion to the parental phenotype (19). However, we never observed the reversion of N315 L3 SCV to the characteristic light golden colonies of the parental strain (Fig. 4C).

*S. aureus* SCVs have been described in clinical settings for more than a century and are strongly associated with chronic infections and resistance to antibiotics (21). However, there is still a major gap in understanding their native properties and the mechanism of SCV phenotype switching. SCV formation may occur through mutations of genes involved in the biosynthesis of thiamine, menadione, hemin, and thymidine, resulting in defects in the electron transport chain and ATP synthesis (21, 24). Alternatively, the formation of SCV may occur via mutations in global regulators or changes in the expression of non-coding RNAs (25). Recently, the molecular mechanism underlying the generation of some unstable SCV was associated with large chromosomal rearrangements (26, 27).

N315 L3 isolates accumulated several mutations during the passaging experiment but only the missense mutations 511G > A *corA* and 259G > A *rsbW* genes acquired at passage 4 became fixed in all subsequent passages (Fig. 2A, D, and 4B). *corA* encodes for a magnesium/cobalt transporter (63) and *rsbW* for an anti-SigB factor (64). *corA* has not been reported to be involved in *S. aureus* virulence or SCV formation, but *S. aureus* preferentially colonizes tissues with high magnesium concentrations (65), which influences biofilm formation and induces a SigB-dependent stress response (66). In a study where biofilms were induced in Mg^2+^-rich solid agar media, the authors observed a switch from orange to white colony phenotype that was dependent on SigB loss of function (67).

Increased SigB expression has been reported for *S. aureus* SCV, and SigB is considered a key regulator of virulence for SCV formation (23, 68–70). *S. aureus* deletion mutants deficient in *sigB* did not generate SCV and were completely cleared by host cells within a few days (68). The activity of SigB is controlled by proteins encoded by the *rsb* operon (*rsbUVWsigB*). RsbW binds to SigB and prevents its binding to the RNA polymerase. In turn, RsbW can be sequestered by the dephosphorylated RsbV, freeing and activating SigB. RsbV is dephosphorylated by RsbU and phosphorylated by RsbW, creating the multiple-step control of this *S. aureus* gene regulator. Mutations in *rsbU* have been reported to lead to the formation of stable, white SCV in continuous *S. aureus* culture in nutrient-rich media (71).

To test the influence of the *corA* and *rsbW* mutations on the N315 L3 SCV phenotype, we constructed isogenic mutants of the WT strain as described in the Materials and Methods. The introduction of the *corA* mutation into the N315 WT background (N315::corA^L3^) did not alter the colony phenotype of the parental strain. Despite many attempts, it was not possible to introduce the *rsbW* mutation into the WT background or the N315::corA^L3^ mutant, suggesting a deleterious effect of the mutation. Therefore, we changed our strategy to reverse the mutations in the N315 L3 evolved strain back to the WT alleles. The reversion of the *rsbW* gene alone resulted in a restoration of the colony phenotype to non-SCV with the same levels of carotenoid production as the parental strain (Fig. 4C). This is, to our knowledge, the first description of an *rsbW* mutation associated with the formation of SCV. However, *rsbW* mutations with distinct phenotypic effects have been reported including a switch from orange to white colonies linked to a SigB-defective phenotype in magnesium-enriched conditions (67). By contrast, a study examining the effects of teicoplanin stress in a natural *rsbU* mutant identified several mutations in *rsbW* that led to an increased SigB activity and increased carotenoid production (72). Taken together, we have identified a novel SCV variant characterized by a hyper-pigmented unstable colony phenotype resulting from a mutation in *rsbW*.

### Missense mutation in *rsbW* leads to the formation of SCV and inactivation of SigB restores growth fitness to N315 L3 isolates

To investigate the genetic basis for the conversion of the orange-SCV to white colonies, we carried out long read-sequencing by PacBio to enable the detection of chromosomal rearrangements, and to characterize the methylome profiles of the different variants. The *S. aureus* SCV genome inversion described by Guérillot et al. occurs through the recombination of inverted copies of the *hsdMS* loci of a type I restriction-modification system, suggesting the possible creation of alternative recognition motifs and DNA methylation patterns depending on the site of inversion (26). Although the role of DNA methylation in *S. aureus* SCV formation is unknown, shifts in methylation between non-SCV and stable SCV have been suggested (71) and epigenetic modifications have been shown to affect gene regulation in several bacterial species, leading to genetically identical bacteria with distinct phenotypes (73).

**Fig 5:**
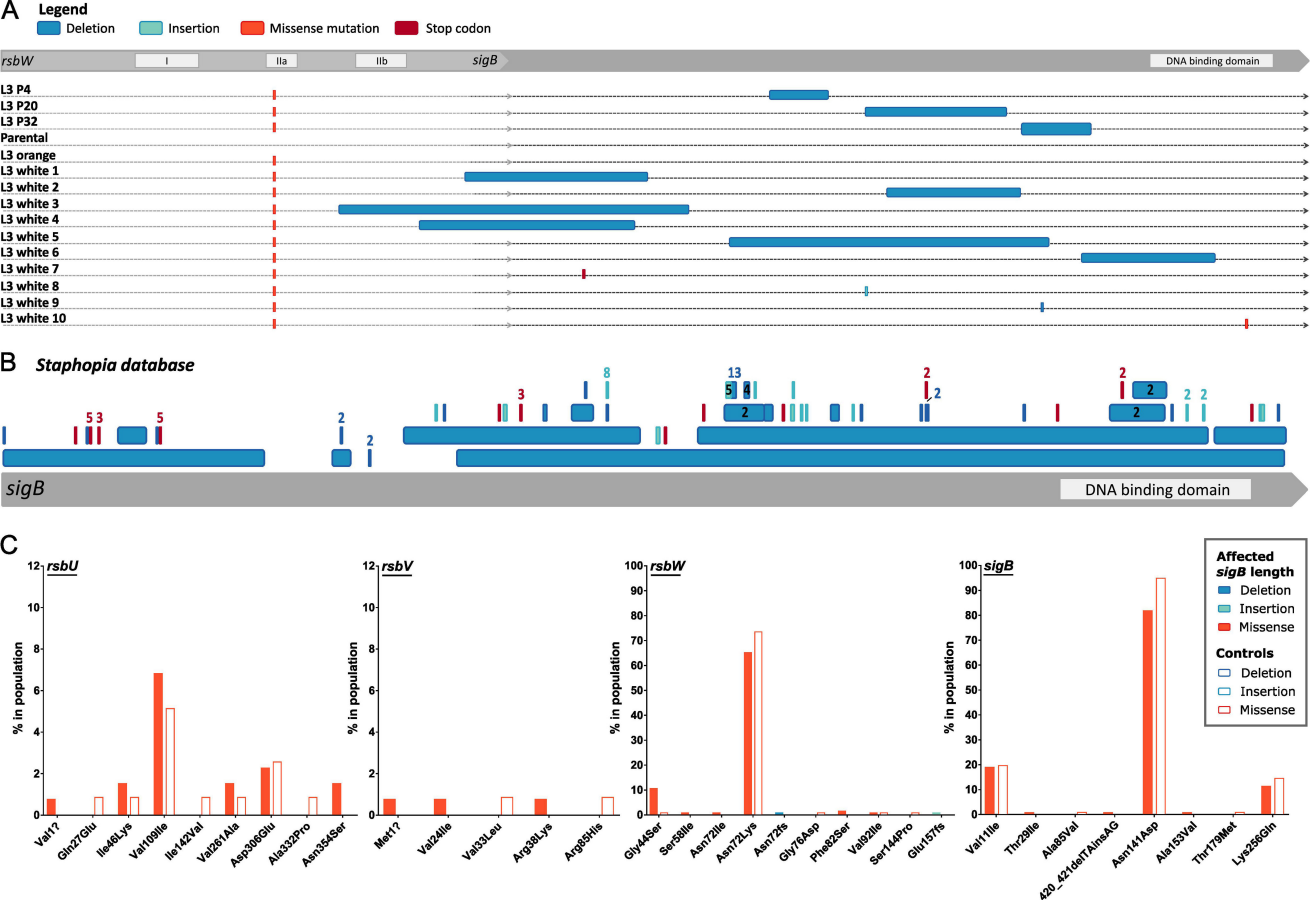
Loss of SCV phenotype in N315 L3 isolates associated with SigB inactivation. (**A**) Inactivation of *sigB* was detected in isolates from passage cycles 4, 20, and 32, and in single L3 white isolates. (**B**) Identification of *sigB* truncations and deletions among publicly available *S. aureus* sequences. Representation of deletions (dark blue), insertions (light blue), and early-stop codons (red) in the 134 *S*. *aureus* genomes from the Staphopia database with changes in *sigB* length. The mutation is numbered when identified more than once. (**C**) Identification of mutations in the *sigB* operon among clinical *S. aureus* isolates exclusively associated with SigB-inactivation. Frequency of specific mutations in *sigB* operon genes among *S. aureus* clinical isolates with a SigB-inactivation (as in 5B) compared with 118 controls.

Chromosomal rearrangements were not observed in either N315 L3 white or orange colonies when compared with the parental strain for both chromosomal and plasmid DNA, and there was no difference in the methylation profile between the sequenced isolates (Fig. S4). However, we identified a deletion of 168 bp in *sigB* among bacteria from a white colony compared to the WT and hyper-pigmented SCV colonies. Sequencing of *sigB* from WT, hyper-pigmented SCV, and white colonies revealed that all white colonies contained disruptive mutations in the *sigB* gene (Fig. 5A). Different types of disruptive mutation were identified including five distinct large deletions (from 123bp to 322 bp), one non-sense mutation, a 1 bp frameshift deletion, and a missense mutation in the DNA binding site of SigB (Fig. 5A). No other mutations were identified between the hyper-pigmented SCV and the white colonies.

The *rsbW* mutation is located in the IIa kinase domain of RsbW, likely disrupting the ability to phosphorylate RsbV, meaning RsbW would remain associated with RsbV, leaving SigB free and active. Since SigB positively regulates genes involved in staphyloxanthin synthesis, this would promote carotenoid production leading to the hyper-pigmented phenotype (Fig. 4B). A *de novo*-designed antimicrobial peptide with inhibitory activity for *S. aureus* RsbW was shown to affect the transcription of genes involved in pigmentation (74), and *S. aureus* N315 that overexpresses SigB was demonstrated to have thickened cell walls, high carotenoid content and reduced susceptibility to antimicrobial peptides and cell wall-tropic antibiotics (75).

### The novel SCV variant promotes survival in macrophages and human blood, and enhanced resistance to vancomycin

To examine the effect of the *rsbW* mutation on bacterial fitness, we compared growth in solid nutrient-rich media, intracellular survival in macrophages, survival in human blood, colonization in a zebrafish embryo model, and antibiotic resistance of the orange-SCV and white N315 L3 with the parental strain. Orange-SCV had an increased ability to survive inside macrophages when compared with N315 L3 white and the parental strain despite exhibiting a growth defect in solid nutrient-rich media (Fig. 4C and D). In fact, the white N315 L3 SCV-revertants had reduced macrophage intracellular survival when compared to the WT strain, consistent with the loss of *sigB* function. Previously, it has been demonstrated that SigB-deficient mutants do not generate SCV and are rapidly cleared by host cells (68). Reversion of the *rsbW* mutation to the WT allele in the N315 L3 strain resulted in a loss of the enhanced growth phenotype. In addition, the *rsbW* mutation increases the survival of *S. aureus* in human blood, and this effect is independent of *sigB* as both orange and white N315 L3 colonies had similar phenotypes compared to the parental strain and the *rsbW* reversion mutant (Fig. 4). Furthermore, the orange SCV also exhibits an intermediate vancomycin resistance phenotype (Fig. 4F), most likely due to an increased thickness in the cell wall (75). Finally, while there was no difference in embryo survival in a zebrafish infection model, the N315 L3 white strain resulted in lower *in vivo* growth levels than the orange-SCV or the parental strain (Fig S5; Fig. 4E), confirming that the inactivation of *sigB* that restored the fitness of the SCV mutants in solid nutrient-rich media led to reduced virulence *in vivo* (Fig. 4). Taken together, these findings highlight the potential clinical relevance of the adaptive mutations identified using the experimental evolution approach *in vitro*.

### Identification of *sigB* truncations and deletions among publicly available *S. aureus* sequences

This is the first study to report a mutation in *rsbW* associated with hyper-pigmented SCV formation followed by conversion to normal-size unpigmented colonies associated with large deletions in *sigB*, but the relevance of this novel type of *S. aureus* SCV to clinical disease in humans is unknown. We searched *S. aureus* genome sequences in the public sequence databases but did not identify the same mutation in *rsbW* that was identified in the current study. Therefore, we interrogated the database for *S. aureus* sequences with truncated *sigB* genes that would be consistent with compensatory genetic events that occurred during the culture of clinical isolates. Among 45,887 *S*. *aureus* genomes deposited in the Staphophia database, we identified 134 *S*. *aureus* genomes with changes in *sigB* length due to indels or non-sense mutations (Fig. 5B; Table S3). Of note, this approach excludes disruptive missense mutations in *sigB* as a mechanism for SigB silencing, similar to the one observed in our study (Fig. 5A, white 10) and in other studies (67, 76). Of the 134 sequences, 103 had *sigB* truncated by frameshifts and 13 were caused by deletions that range from 11 to 498 bp (Fig. 5A). Previous studies have reported 2 bp and 163 bp natural deletions that resulted in *sigB* disruption, the first identified during cloning and the second in a study examining biofilm formation (72, 76). To verify whether there is an association between disruptions in *sigB* function and mutations in the *rsbUVWsigB* operon we have compared the allelic variation of the *rsbUVWsigB* operon in strains with and without disruptions in the *sigB* gene. Among sequences in the database with truncated *sigB* genes, there was considerable allelic variation in *rsb* genes with 119 mutations in *sigB* operon genes (Table S4). Of these, mutations in 1G > A, 1061A > G in *rsbU*; 2T > C, 70G > A, 113G > A in *rsbV*; 173G > T, 215A > T, 245T > C, 216delT, 468dupA in *rsbW*; 86C > T, 420_421delTAinsAG, 458C > T in *sigB* were found exclusively in strains that contained a truncated *sigB* in addition to a 130G > A mutation in *rsbW* that was found in a higher percentage compared to control strains (Fig. 5C; Tables S4 and S5). The existence of *S. aureus* genome sequences from the public databases with *sigB* truncations, along with corresponding variation in *rsb* gene sequences, suggests that related SCV phenotypes associated with subsequent loss of *sigB* function during culture may occur during infection, warranting further investigations to assess the clinical relevance of this phenomenon.

### Conclusions

Taken together, we have developed an evolutionary model of *S. aureus* macrophage infections and identified a novel SCV variant phenotype that promotes survival in macrophages, whole blood, and in the presence of vancomycin. Importantly, our findings suggest that models of evolution that incorporate interactions with innate immune cells may be useful for revealing clinically relevant adaptive traits that are not readily recovered from clinical samples due to lack of viability or negative selection during growth in nutrient-rich media. Our data provide new insights into the capacity of *S. aureus* to adapt to different niches during infection.

## Data Availability

Sequence data have been deposited in the European Nucleotide Archive (ENA) database under Bioproject number PRJEB72281. Accession numbers of each genome are listed in Table S6.

## SUPPLEMENTAL MATERIAL

Supplemental Figures mbio.00346-24-s0001.docx Fig. S1-S5.

Supplemental Tables mbio.00346-24-s0002.xlsx Tables S1-S6.

ASM does not own the copyrights to Supplemental Material that may be linked to, or accessed through, an article. The authors have granted ASM a non-exclusive, world-wide license to publish the Supplemental Material files. Please contact the corresponding author directly for reuse.
