# Supplementary material for: Experimental evolution of Staphylococcus aureus in macrophages: dissection of a conditional adaptive trait promoting intracellular survival
Source: mBio. 2024 Apr 29;15(6):e00346-24. doi: 10.1128/mbio.00346-24 (PMC11237485; doi:10.1128/mbio.00346-24)
Supplement: Supplemental Figures — Fig. S1-S5. [file mbio.00346-24-s0001.docx]

**Figure S1**


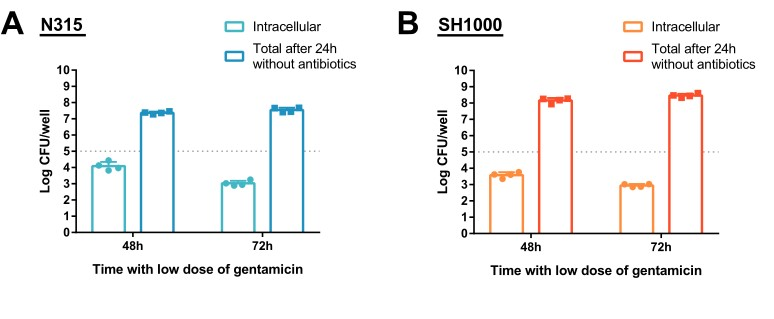


**Figure S1. Bacterial numbers recovered from inside macrophages is insufficient for a new re-infection cycle.** Number of CFU recovered from inside THP-1 macrophages infected for 1h with (A) N135 or (B) SH1000 at MOI 1, followed by a 48h or 72h treatment with 10µg/ml of gentamicin and total amount of bacteria recovered 24h after the removal of the antibiotic. Dotted line represents the amount of bacteria needed for a new re-infection cycle. Graphic representative of an independent experiment. Each symbol represents a technical replicate.

**Figure S2**


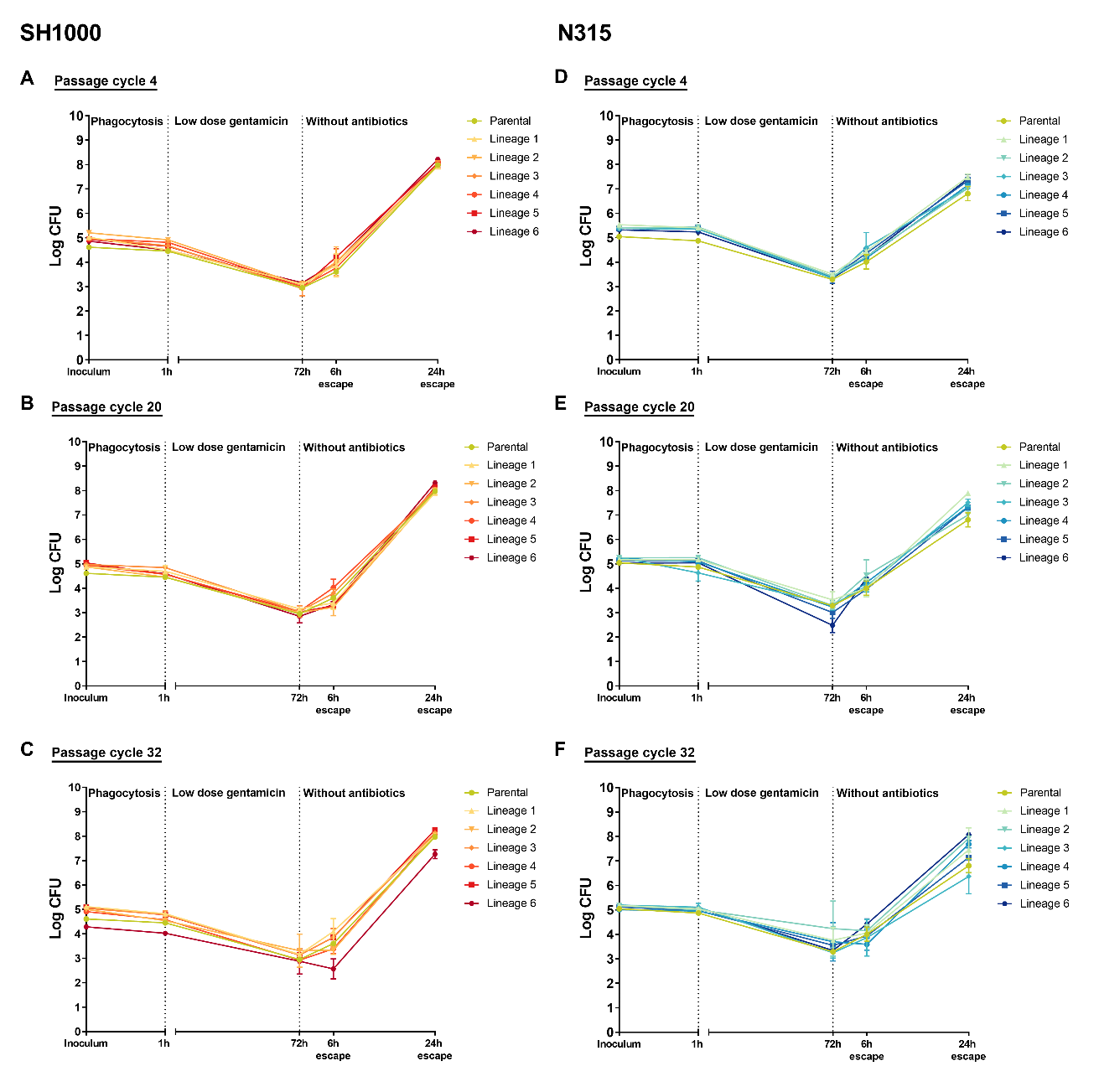


**Figure S2. Macrophage-passaged *S. aureus* variants exhibit a similar level of macrophage survival compared to the parental strain when grown in TSB prior to infection.** Parental strain and isolates from passage cycles 4 (A and D), 20 (B and E) and 32 (C and F) were grown in TSB to exponential phase and THP-1 PMA-differentiated macrophages were infected at MOI of 1. After 1 hour of infection, the media was replaced with media with 10 µg/ml of gentamicin for 72h. Bacterial escape from the macrophage intracellular environment and growth in the media was evaluated 6 and 24h after removal of the antibiotics. To evaluate the bacterial levels at the different time points, macrophages were lysed with 0.01% Triton X-100. Data for (A, C and E) SH1000 and (B, D and F) N315 infections.

**Figure S3**


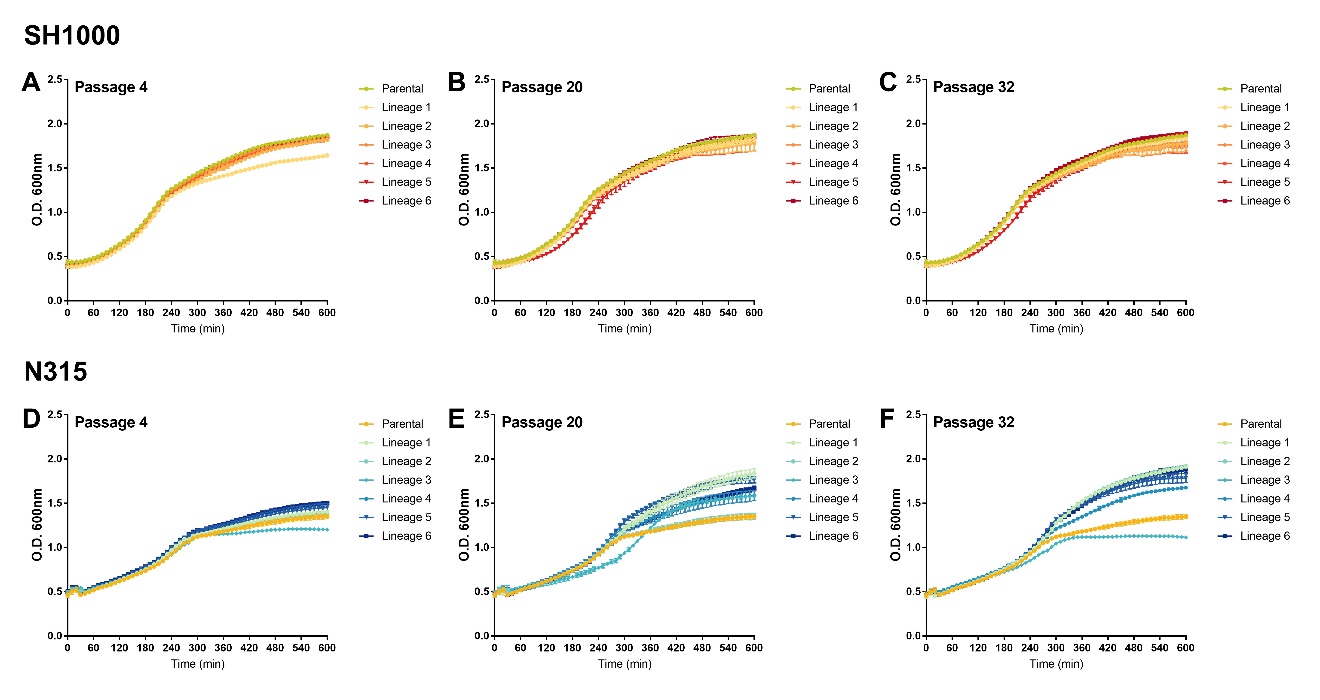


**Figure S3. *S. aureus* N315 isolates from the evolution assay exhibit increased growth in macrophage cell culture media compared to the parental strain.** Growth curves of parental strain and isolates from passage cycles 4 (A and D), 20 (B and E) and 32 (C and F) in macrophage cell culture media at 37°C. Data for (A, C and E) SH1000 and (B, D and F) N315 strains.

**Figure S4**


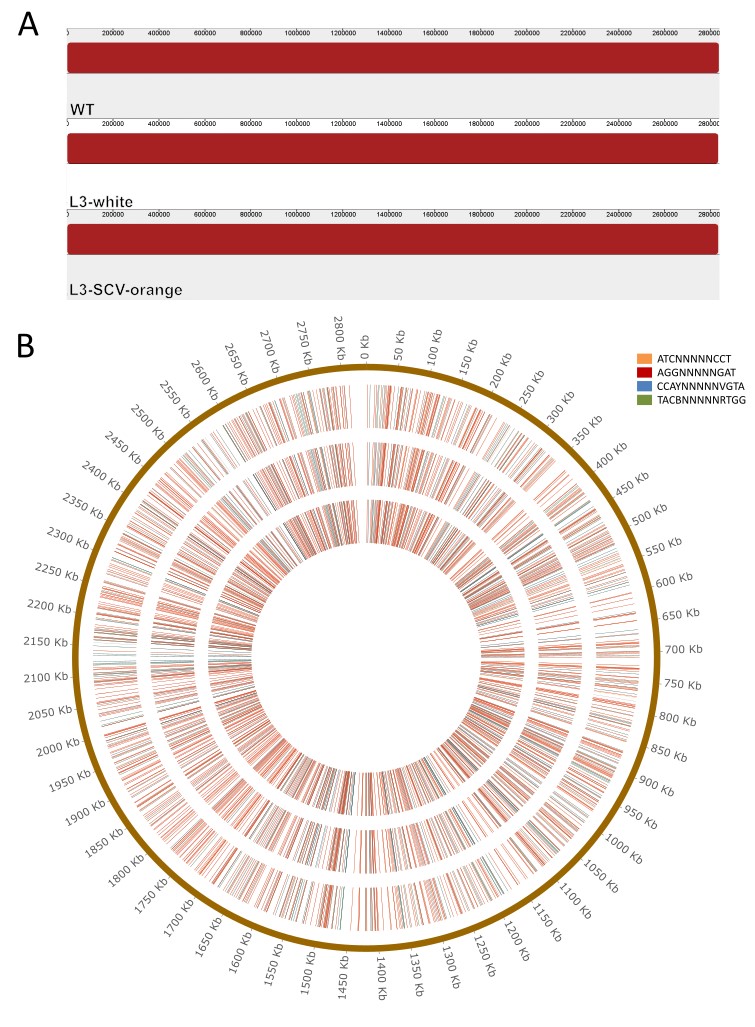


Figure S4. Lack of chromosomal rearrangements or changes in methylation patterns between Parental, L3 white and L3 orange isolates. (A) progressiveMauve alignments of closed genomes from the parental N315 strain (sample 1), L3 white (sample 2) and L3 orange (sample 3) colony producing passaged isolates. Genomes were assembled from 200x coverage of PacBio long reads using Flye. (B) Circos plot of the methylation patterns on parental N315 strain (outer circle), L3 white (middle circle) and L3 orange (inner circle). Methylation detected by PacBio.

**Figure S5**


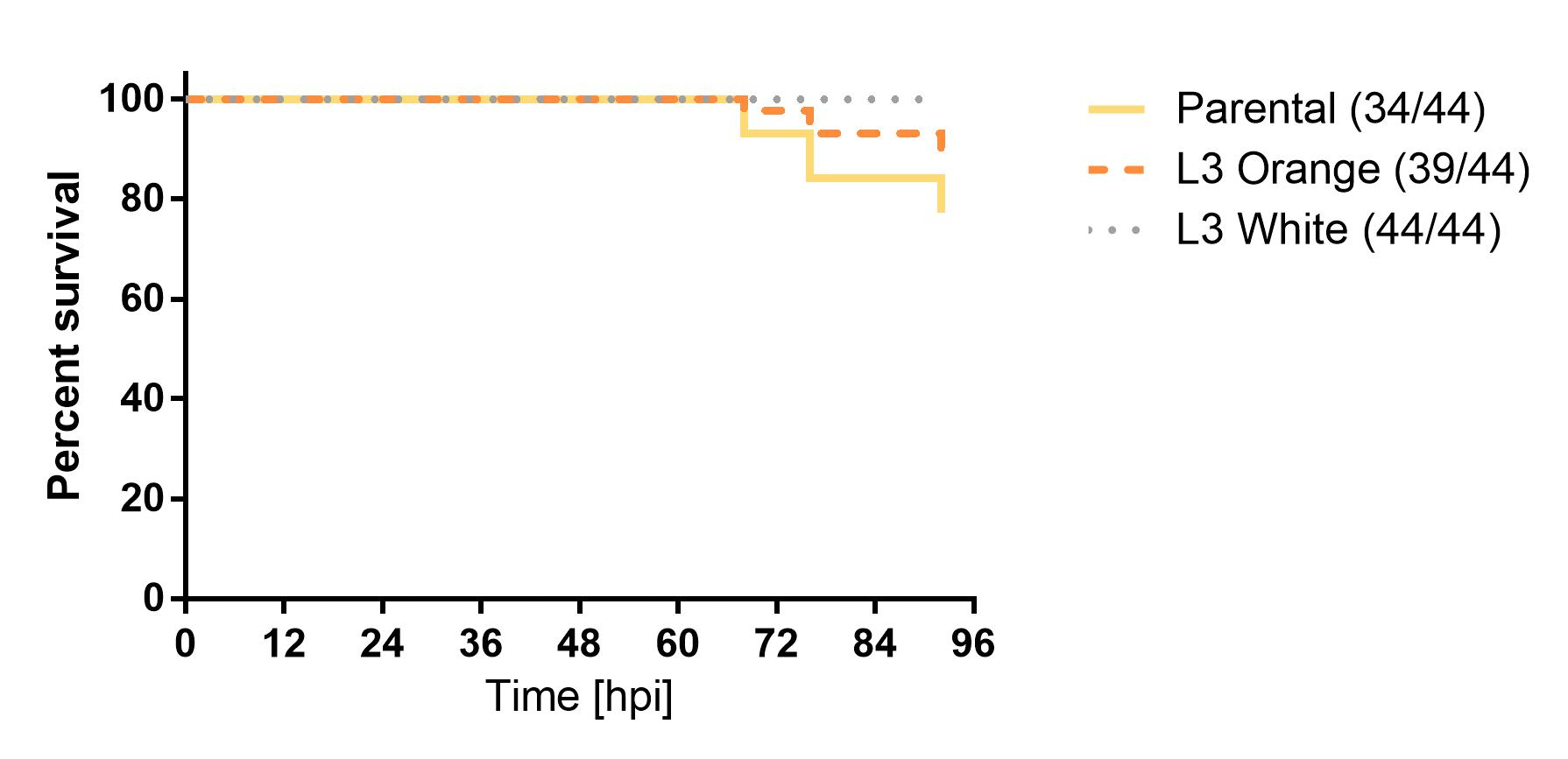


Figure S5. Zebrafish embryo survival after infection with Parental, L3 white and L3 orange isolate. 30 hours post-fertilization WT zebrafish embryos infected intravenously with 3-3.5x10^3^ CFU *S. aureus* N315 isolates. Combination of two independent survival experiments. In parenthesis the number of live embryos / total infected embryos at 92h post infection.
